# Optical Coherence Tomography in Alzheimer’s Disease: A Meta-Analysis

**DOI:** 10.1371/journal.pone.0134750

**Published:** 2015-08-07

**Authors:** Gianluca Coppola, Antonio Di Renzo, Lucia Ziccardi, Francesco Martelli, Antonello Fadda, Gianluca Manni, Piero Barboni, Francesco Pierelli, Alfredo A. Sadun, Vincenzo Parisi

**Affiliations:** 1 G.B. Bietti Foundation-IRCCS, Department of Neurophysiology of Vision and Neurophthalmology, Rome, Italy; 2 Istituto Superiore di Sanità, Dipartimento Tecnologie e Salute, Rome, Italy; 3 Tor Vergata University of Rome, Department of Clinical Sciences and Translation Medicine; Rome, Italy; 4 IRCCS Istituto Scientifico San Raffaele, Milan, Italy; 5 Sapienza University of Rome Polo Pontino, Department of Medico-Surgical Sciences and Biotechnologies, Latina, Italy; 6 IRCCS-Neuromed, Pozzilli (IS), Italy; 7 Doheny Eye Institute, Keck School of Medicine, University of Southern California, Los Angeles, California, United States of America; University Of São Paulo, BRAZIL

## Abstract

**Background:**

Alzheimer’s disease (AD) is a neurodegenerative disorder, which is likely to start as mild cognitive impairment (MCI) several years before the its full-blown clinical manifestation. Optical coherence tomography (OCT) has been used to detect a loss in peripapillary retina nerve fiber layer (RNFL) and a reduction in macular thickness and volume of people affected by MCI or AD. Here, we performed an aggregate meta-analysis combining results from different studies.

**Methods and Findings:**

Data sources were case-control studies published between January 2001 and August 2014 (identified through PubMed and Google Scholar databases) that examined the RNFL thickness by means of OCT in AD and MCI patients compared with cognitively healthy controls.

**Results:**

11 studies were identified, including 380 patients with AD, 68 with MCI and 293 healthy controls (HC). The studies suggest that the mean RNFL thickness is reduced in MCI (weighted mean differences in μm, WMD = -13.39, 95% CI: -17.34 to -9.45, p = 0.031) and, even more so, in AD (WMD = -15.95, 95% CI: -21.65 to -10.21, p<0.0001) patients compared to HC. RNFL in the 4 quadrants were all significantly thinner in AD superior (superior WMD = -24.0, 95% CI: -34.9 to -13.1, p<0.0001; inferior WMD = -20.8, 95% CI: -32.0 to -9.7, p<0.0001; nasal WMD = -14.7, 95% CI: -23.9 to -5.5, p<0.0001; and temporal WMD = -10.7, 95% CI: -19.9 to -1.4, p<0.0001); the same significant reduction in quadrant RNFL was observed in MCI patients compared with HC (Inferior WMD = -20.22, 95% CI: -30.41 to -10.03, p = 0.0001; nasal WMD = -7.4, 95% CI: -10.08 to -4.7, p = 0.0000; and temporal WMD = -6.88, 95% CI: -12.62 to -1.13, p = 0.01), with the exception of superior quadrant (WMD = -19.45, 95% CI: -40.23 to 1.32, p = 0.06).

**Conclusion:**

Results from the meta-analysis support the important role of OCT for RNFL analysis in monitoring the progression of AD and in assessing the effectiveness of purported AD treatments.

## Introduction

Amongst the various possible causes of dementia, Alzheimer’s disease (AD) is the most common with an incidence that exponentially increases with age. AD is a brain degenerative disorder, where complex relationships between inherited susceptibility and environmental factors may play roles [1,2]. Because of the slowness of the disease’s progression, the neurodegenerative processes are likely to start many years before the full-blown clinical manifestation of AD. This variable transitional phase is clinically recognized as a separate entity, mild cognitive impairment (MCI) [3]. Cognitive and functional severity within the MCI definition varies over a wide range, so that the syndrome of MCI is not clinically homogeneous [4]. Although subjects with MCI have an increased risk of progressing to dementia, most remain stable or return to normality [5].

Despite great advances in the understanding of AD pathophysiology in the last few years, the exact pathogenesis of AD and of its precursor MCI are still not comprehensively understood.

Various visual disturbances may affect AD patients [6–13], which have been historically attributed to damage and/or degenerative processes in primary and associative visual cortical areas [14–18]. However, during the last few decades, some authors realized that cortical dysfunctions alone cannot explain entirely the pattern of observed defects [19]. Specifically, multiple forms of evidence points toward the involvement of retinal ganglion cells and their axons in the optic nerve as a basis of the visual dysfunction in AD [20,21]. In fact, histopathological lesions associated with AD—neuronal loss, beta-amyloid plaques, neurofibrillary tangles, and granulovacuolar degeneration—have been seen not only in brain structures historically thought to be involved in AD [15,16], but also within the neuroretina [20,22].

In the last two decades, several studies have searched for in vivo evidence of the retinal involvement in AD pathophysiology. Sophisticated imaging techniques have been used, including Optical Coherence Tomography (OCT) which has been extensively used to assess the morphological changes of the retina in AD and other dementing disorders. OCT permits the objective quantification *in vivo* of the retinal nerve fiber layer (RNFL) that consists of axons that form the optic nerve axons and contributes partially to the retinal thickness. This method consists of a non-invasive technology that allows for imaging of the eye [23,24]. Taking in mind that the human eye is an embryological protrusion of the brain, and the nerves and axons of the RNFL is a tract of the brain, it is not surprising that OCT has been widely employed in assessing RNFL thickness in several neurological disorders [25–30]. The OCT technique for the measurement of the peripapillary RNFL, the macular thickness and volume, has been proven useful for the detection of significant reduced retinal thickness in patients with AD [31–36] and in those affected with MCI [37]. Because early diagnosis of AD remains a big challenge, since up to now can be definitively confirmed only with post mortem histopathology, the discovery of new non-invasive in vivo biological markers is a major aim in current research on AD [38]. In this context, reduced retinal thickness measured with OCT may be a promising biomarker for monitoring progression from normal and abnormal age-related processes, such as for instance supranuclear cataract and opacities of ocular lens, to the pathological neural degeneration undoubtedly associated with MCI and AD [39]. However, an aggregate analysis combining results from different studies is lacking. It is thus of particular interest to assess whether the retinal morphological changes may be related to cognitive impairment. Only a few published works have attempted correlations between the loss in RNFL and the neuropsychological indexes of cognitive impairment reported so the results are inconclusive [40–44].

The intent of this study is to provide a comprehensive meta-analysis overview of the available results provided by the OCT technique as used to understand morphological retinal changes that occur due to the degenerative processes associated with AD and MCI.

## Materials and Methods

We followed methodology already published elsewhere [45]. In performing this meta-analysis we followed the PRISMA (Preferred Reporting Items for Systematic Review and Meta-Analyses) statement (S1 Fig) [46]. We initially searched the PubMed database to identify articles published up to August 2014. The search terms used were “Alzheimer’s disease”, “Dementia”, “Optical coherence tomography”, and “retinal nerve fiber layer”, alone and in combination. The literature search was updated using the additional keywords “Alzheimer’s disease”, “electroretinogram” and “visual evoked potentials” to identify full-text papers written in English and published in peer-reviewed journals up to August 2014, using the PubMed and Google Scholar databases. In addition, we manually searched the reference lists of all primary articles and review articles. The initial search identified 14 articles (S1 Fig).

### Inclusion and exclusion criteria

For inclusion in the meta-analysis a study had to meet the following criteria: (1) case-control or cross-sectional design; (2) OCT data were reported as mean or standard deviation; (3) AD and MCI patients were diagnosed according to established diagnostic systems (DSM-III, DSM-IV, ICD 9, ICD 10); (4) studies should provide the data of peripapillary and/or macular RNFL thickness; and (5) sample size ≥9 in each group. (5) Only published studies were included, abstracts were ignored.

We used a two-step selection processes to identify eligible studies. In the first step, two investigators (GC, VP) screened the title and abstracts and by consensus identified all studies that did not meet any of the prespecified criteria. We excluded these studies. In the second step, the same investigators evaluated the full text versions of the remaining studies. Studies were excluded if they did not meet all criteria.

### Data extraction

Two investigators (GC, LZ) independently extracted data and entered them in a customised database. Disagreements were resolved by consensus. The extracted data included authors and title of study, year of publication, study design, study size, study participants (AD, MCI, controls), mean age of the participants, severity of the disease (as assessed by MMSE or as classified in mild, moderate or severe AD), and OCT apparatus type. The parapapillary RNFL thickness parameters evaluated in these studies were average thickness (360° measurement), temporal quadrant thickness (316–45°), superior quadrant thickness (46–135°), nasal quadrant thickness (136–225°) and inferior quadrant thickness (226–315°). All data were extracted from the published studies and we did not contact the authors for further information.

### Statistical analysis

Original data were obtained from the articles as much as possible. Data that could not be obtained were calculated when necessary. When standard deviation (SD) was not available, it was calculated using the sample sizes, standard error or, if not available, by extrapolating data from the bar chart. Statistical analysis was performed using custom written software in Mat-Lab environment (www.mathwork.com) and R statistical software (The R Foundation for statistical Computing v3.1.2). Summary estimates, including 95% confidence intervals (CIs), were calculated. For continuous outcome, means and standard deviations were used to calculate the weighted mean difference using a random-effects model (WMD). The chi-square test, tau^2^ and the Higgins I^2^ test were used to assess heterogeneity [47]. The I^2^ test is a method for quantifying inconsistency across studies and describes the percentage of variability in effect estimates that is due to heterogeneity. A value greater than 50% was considered as substantial heterogeneity. If there was no heterogeneity across studies (P>0.1, I^2^,50%), we adopt fixed-effects model for analysis. Otherwise, random-effects model was used. Potential publication bias was examined using a funnel plot [48]. A P value less than 0.05 was considered statistically significant. A strong correlation between sample size and summary estimates suggests publication bias.

## Results

**Fig 1:**
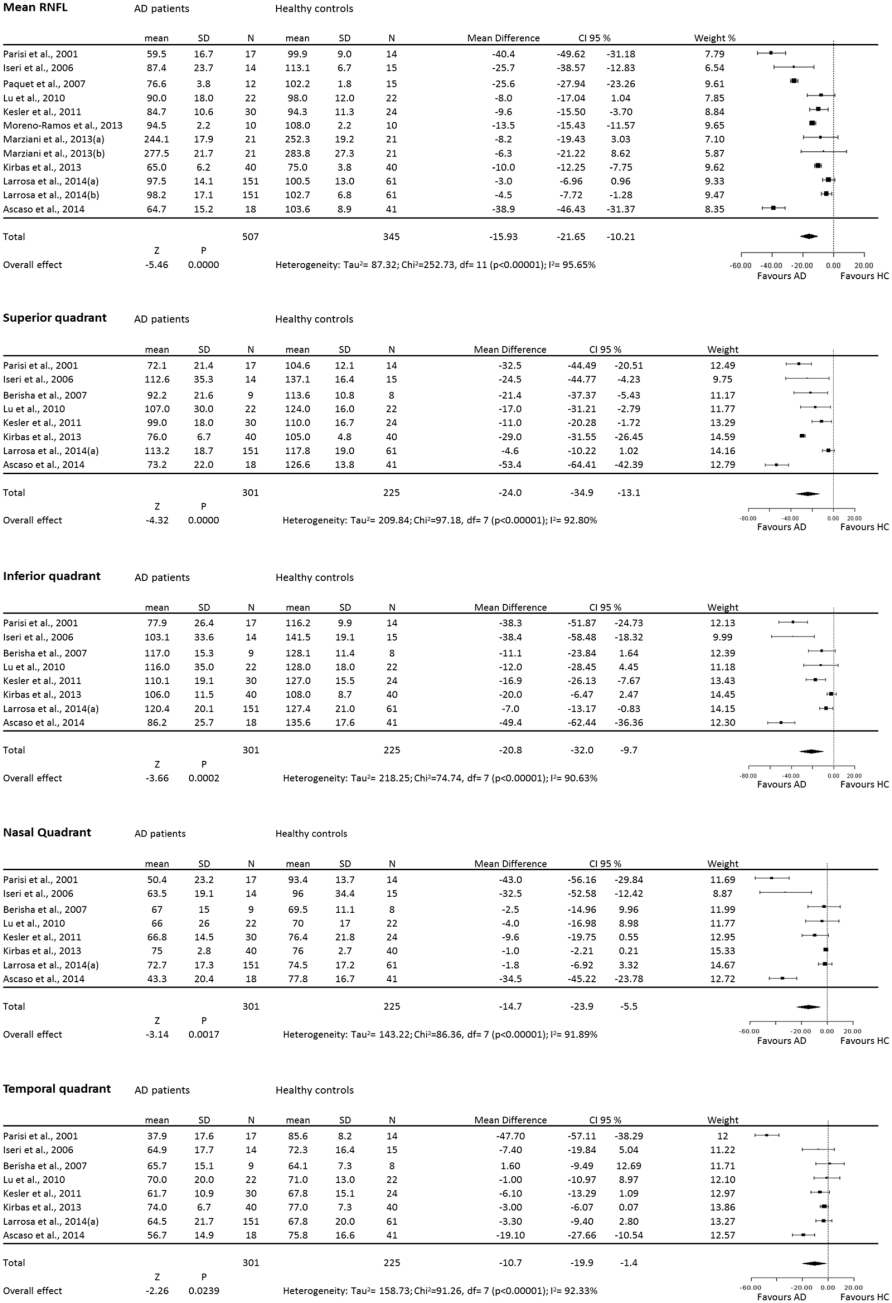
Forest plots of weighted mean difference (WMD) of AD patients for the mean and each single quadrant RNFL. Horizontal lines are 95% confidence intervals.

**Fig 2:**
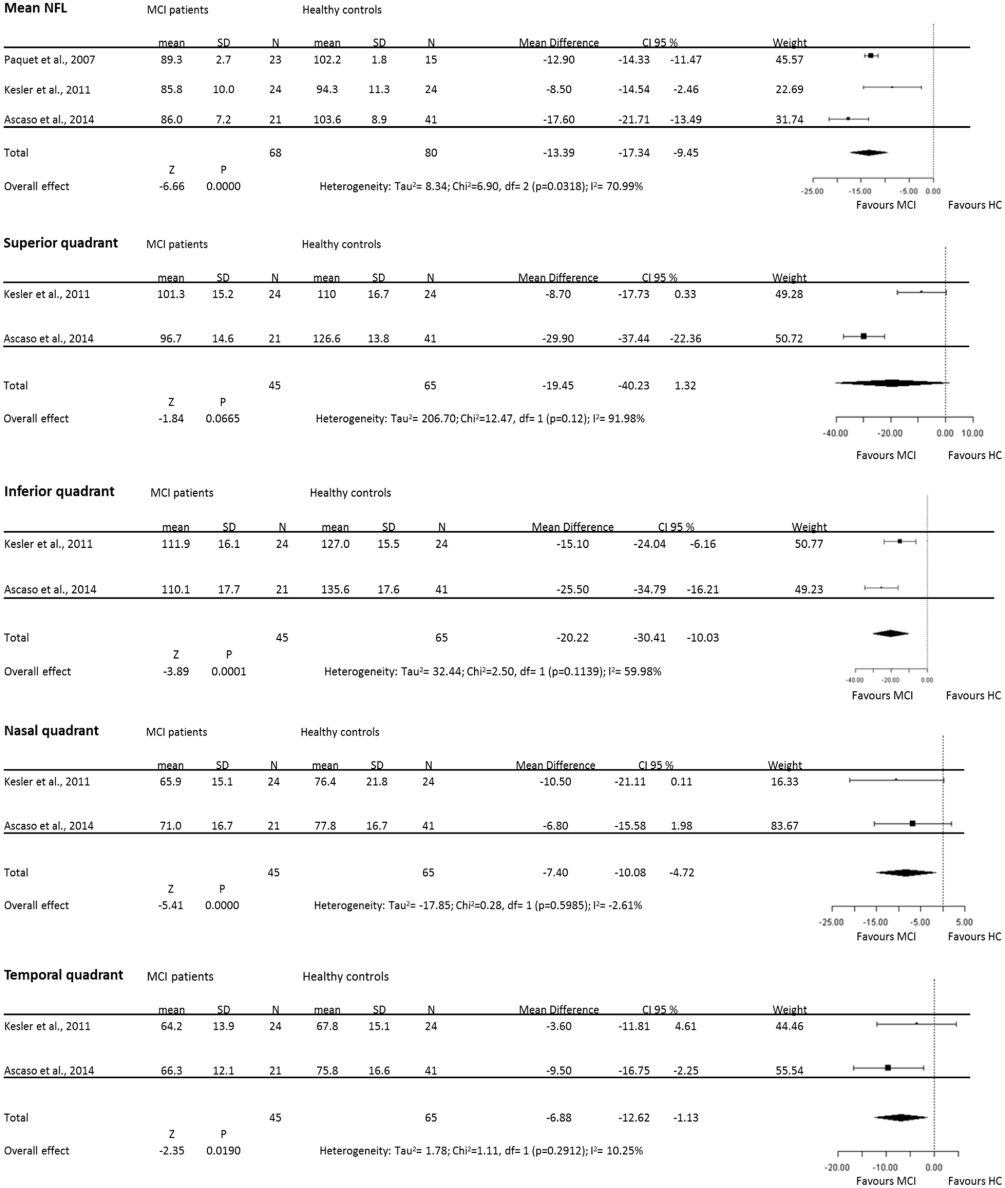
Forest plots of weighted mean difference (WMD) of MCI patients for the mean and each single quadrant RNFL. Horizontal lines are 95% confidence intervals.

A total of 14 articles were initially identified; 2 articles were excluded due to duplications, and 1 article due to lack of data. We identified 11 articles on OCT in AD, of which 3 also contained data inMCI, that were suitable for analysis (see Table 1). However, 2 out of 11 articles were considered twice [36],[49] in the analysis due to the fact that the participants were scanned twice, with different OCT apparatus type. Overall, from the 11 studies we included a total of 380 patients with AD, 68 with MCI and 293 healthy controls. For the mean RNFL we identified 10 studies suitable for analysis, which included 349 AD patients, 68 MCI patients, and 263 healthy volunteers. For the quadrant RNFL we identified 8 studies suitable for analysis, which included 301 AD patients, 45 MCI patients, and 225 healthy controls. The articles included are described in Table 1 and the main results of the meta-analysis are summarised in Figs 1 and 2.

**Table 1:** Demographic data and retinal NFL thickness measurements in patients and controls as determined by OCT.

Reference	No. Of subjects and diagnosis	Mean MMSE/AD stage	Mean age ± SD	OCT machine	Mean NFL (μm)	Superior quadrant (μm)	Inferior quadrant (μm)	Nasal quadrant (μm)	Temporal quadrant (μm)	Note
Parisi et al. [31,32]	17 AD	/mild	70.37 ± 6.1	Humphrey	59.5 ± 16.70*	72.1 ± 21.4*	77.9±26.4*	50.4±23.2*	37.9±17.60*	NFL overall values correlated with PERG
	14 controls				99.9 ± 8.95	104.6 ± 12.1	116.2±9.87	93.4±13.7	85.6±8.21	
Iseri et al. [60]	14 AD	18.5/mild to moderate	70.1 ± 9.7	Carl Zeiss Meditec, Model 3000	87.4 ± 23.7*	112.6 ± 35.3*	103.1 ± 33.6*	63.5 ± 19.1*	64.9 ± 17.7	Decline in both peripapillary and macular thickness and volume in AD eyes
	15 controls	29.4	65.1 ± 9.8		113.1 ± 6.7	137.1 ± 16.4	141.5 ± 19.1	96.0 ± 34.4	72.3 ± 16.4	
Berisha et al. [64]	9 AD	23.8/mild to moderate	74.3 ± 3.3	Carl Zeiss Meditec, Model 3000		92.2 ± 21.6*	117.0 ± 15.3	67.0 ± 15.0	65.7 ± 15.1	Narrowing of the retinal microvasculature
	8 HV	29.5	74.3 ± 5.8			113.6 ± 10.8	128.1 ± 11.4	69.5 ± 11.1	64.1 ± 7.3	
Paquet et al. [33]	23 MCI	28.8	78.7 ± 6.2	Carl Zeiss Stratus OCT 3	89.3 ± 2.7*					The involvement of retina is an early event in the course of this disorder
	14 AD	22.6/mild	78.3 ± 5.1		89.2 ± 2.9*					
	12 AD	16.6/severe	78.8 ± 4.9		76.6 ± 3.8*					
	15 controls	28.9	75.5 ± 5.1		102.2 ± 1.8					
Lu et al. [34]	22 AD		73 ± 8	Carl Zeiss Meditec, Model 3000	90 ± 18*(§)	107 ± 30*(§)	116 ± 35*(§)	66 ± 26	70 ± 20	Enlarged optic cup to disc ratio in AD
	22 controls		68 ± 9		98 ± 12	124 ± 16	128 ± 18	70 ± 17	71 ± 13	
Kesler et al. [37]	24 MCI	28.1	71.0 ± 10.0	Carl Zeiss Stratus OCT 3	85.8 ± 10.0*	101.3 ± 15.2	111.9 ± 16.1*	65.9 ± 15.1	64.2 ± 13.9	RNFL thickness not correlated with MMSE
	30 AD	23.6	73.7 ± 9.9		84.7 ± 10.6*	99.0 ± 18.0*	110.1 ± 19.1*	66.8 ± 14.5	61.7 ± 10.9	
	24 controls		70.9 ± 9.2		94.3 ± 11.3	110.0 ± 16.7	127.0 ± 15.5	76.4 ± 21.8	67.8 ± 15.1	
Moreno-Ramos et al. [61]	10 AD	16.4	73.0 ± 6.5	TOPCON 3D OCT-1000	94.5 ± 2.2*					Retinal involvement measured by OCT may also be present in non-AD dementias
	10 LB	14.9	74.2 ± 5.1		93.3 ± 1.5*					
	10 PD	16.4	74.3 ± 5.0		94.8 ± 2.0*					
	10 controls	29.2	70.2 ± 5.5		108.0 ± 2.2					
Marziani et al. [36]	21 AD	19.9/mild to moderate	79.3 ± 5.7	(1) Optovue RTVue-100	244.1 ± 17.9^1^					Reduced RNFL in AD patients using 2 different OCT instruments
					277.5 ± 21.7^2^					
	21 controls	27.9	77.0 ± 4.2	(2) Spectralis Heidelberg Engineering	252.3 ± 19.2^1^ ^ç^					
					283.8 ± 27.3^2^ ^ç^					
Kirbas et al. [35]	40 AD	21.5	69.3 ± 4.9	Spectral domain OCT	65 ± 6.2*	76 ± 6.7*	106 ± 11.5	75 ± 2.8	74 ± 6.7	No correlation between MMSE and OCT results
	40 controls		68.9 ± 5.1		75 ± 3.8	105 ± 4.8	108 ± 8.7	76 ± 2.7	77 ± 7.3	
Larrosa et al. [49]	151 AD	18.31	75.29	(1) Carl Zeiss Meditec Cirrus	97.5 ± 14.1^1^	113.2 ± 18.7^1^ *	120.4 ± 20.1^1^ *	72.7 ± 17.3^1^	64.5 ± 21.7^1^ *	RNFL measurements were a very useful and precise tool for AD diagnosis.
					98.2 ± 17.1^2^ *					
	61 controls		74.87	(2) Spectralis Heidelberg Engineering	100.5 ± 13.0^1^	117.8 ± 19.0^1^ *	127.4 ± 21.0^1^	74.5 ± 17.2^1^	67.8 ± 20.0^1^	
					102.7 ± 6.7^2^					
Ascaso et al. [44]	21 aMCI	19.3	72.1 (AD+aMCI)	Stratus OCT 3	86.0 ± 7.2* #	96.7 ± 14.6*	110.1 ± 17.7*	71.0 ± 16.7 *	66.3 ± 12.1 *	A significant association between RNFL thickness in superior and nasal quadrants, and MMSE score
	18 AD				64.7 ± 15.2* ** #	73.2 ± 22.0* **	86.2 ± 25.7* **	43.3 ± 20.4 * **	56.7 ± 14.9 * **	
	41 controls	28.8	72.9		103.6 ± 8.9 #	126.6 ± 13.8	135.6 ± 17.6	77.8 ± 16.7	75.8 ± 16.6	

AD, Alzheimer’s disease; aMCI, amnestic mild cognitive impairment; LB, dementia with Lewy bodies; MCI, mild cognitive impairment; MMSE, mini mental state examination; RNFL, retinal nerve fiber layer; PD, dementia associated with Parkinson’s disease;

*, significantly different from controls;

**, significantly different from MCI;

^§^ data extrapolated from the bar chart;

^1^ or ^2^ refer to the corresponding OCT machine;

^ç^ Central sector;

^#^ data showed from the right eye only.

### Meta-analysis of OCT data in AD patients

Analysis of mean RNFL thickness in 11 studies between AD patients and healthy controls found significant heterogeneity (I^2^ = 95.65%) across the studies, so the data were pooled through the random effects model. The meta-analysis of these data showed that the mean RNFL thickness in AD was reduced significantly compared with healthy controls (WMD = -15.95, 95% CI: -21.65 to -10.21, p<0.0001, Fig 1). Moreover meta-analysis of each quadrant data showed that there was overall good heterogeneity across studies and a significant difference of RNFL thickness between the two groups in the all 4 quadrants: superior (I^2^ = 92.80%, WMD = -24.0, 95% CI: -34.9 to -13.1, p<0.0001), inferior (I^2^ = 90.62%, WMD = -20.8, 95% CI: -32.0 to -9.7, p<0.0001), nasal (I^2^ = 91.89%, WMD = -14.7, 95% CI: -23.9 to -5.5, p<0.0001), and temporal (I^2^ = 91.26%, WMD = -10.7, 95% CI: -19.9 to -1.4, p<0.0001) quadrants. In summary, the results of meta-analysis showed that there was a significant RNFL thickness reduction in all quadrants in AD patients compared with the control group.

### Meta-analysis of OCT data in MCI patients

Analysis of mean RNFL thickness in 3 studies between MCI patients and healthy controls found less, but still significant, heterogeneity (I^2^ = 70.99%) across the studies, so the data were pooled through the random effects model. The meta-analysis of these data showed that the mean RNFL thickness in MCI was reduced significantly compared with healthy controls (WMD = -13.39, 95% CI: -17.34 to -9.45, p = 0.031, Fig 2). Moreover meta-analysis of each quadrant data showed that there was, with the exception of the superior quadrant, an overall poor heterogeneity across studies: superior (I^2^ = 91.98%), inferior (I^2^ = 59.98%), nasal (I^2^ = -2.61%), and temporal (I^2^ = 10.25%). However, data showed significant thinner RNFL in the inferior (WMD = -20.22, 95% CI: -30.41 to -10.03, p = 0.0001), nasal (WMD = -7.4, 95% CI: -10.08 to -4.7, p = 0.0000), and temporal (WMD = -6.88, 95% CI: -12.62 to -1.13, p = 0.01), quadrants with the exception of the superior quadrant where RNFL just tent to be thinner respect to HC (WMD = -19.45, 95% CI: -40.23 to 1.32, p = 0.06). In summary, the results of meta-analysis showed that there was a significant RNFL thickness reduction in the mean and in single quadrants RNFL in MCI patients, with the notable exception of the superior quadrant.

### Publication Bias

**Fig 3:**
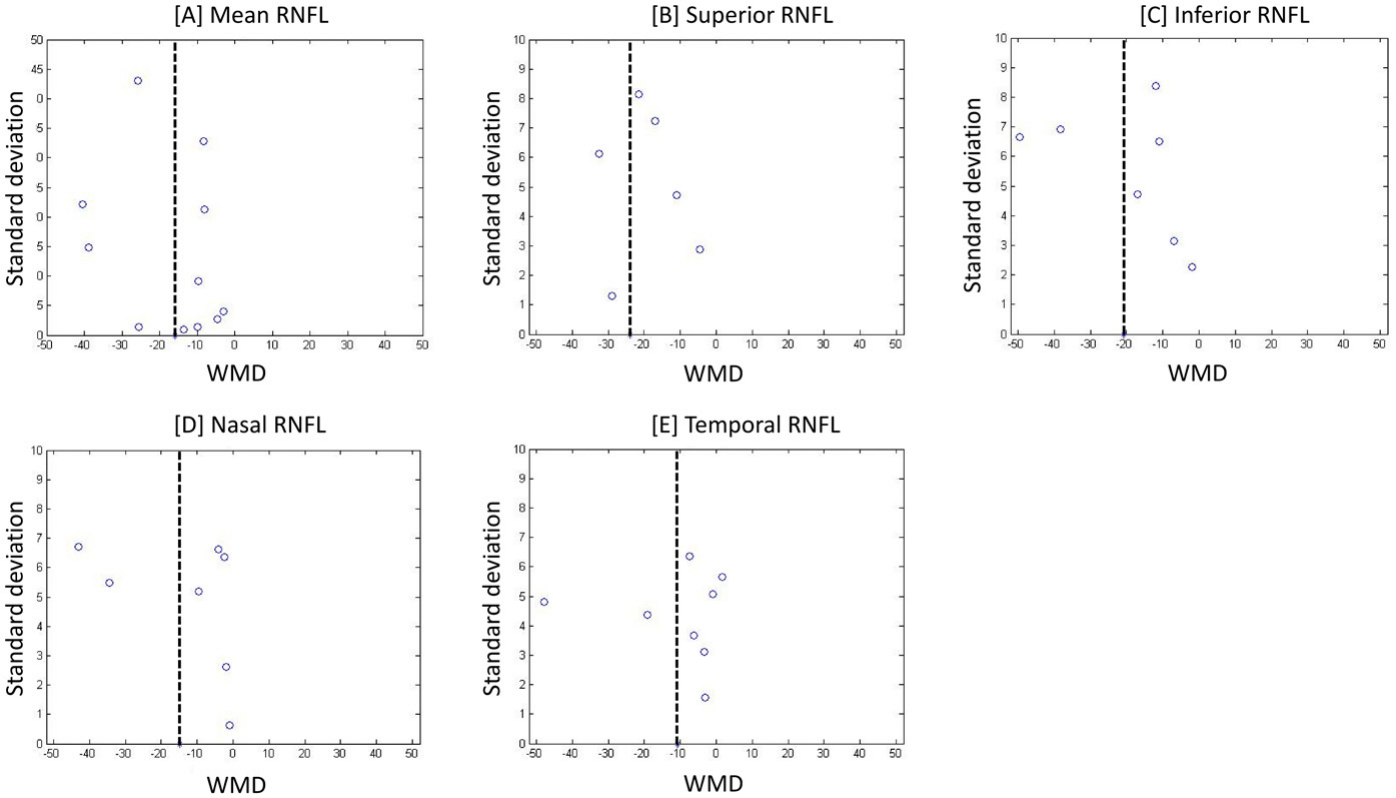
Funnel plots for evaluating the publication bias. Points indicate weighted mean difference (WMD) from studies included in meta—analysis of the mean [A], superior [B], inferior [C], nasal [D] and temporal [E] RNFL quadrants.

From the funnel plot analysis it appears that there is no correlation between study size and effect size or any other evidence of publication bias (Fig 3).

## Discussion

In this review, we performed a comprehensive meta-analysis investigating the role of OCT in detecting reduced RNFL thickness in AD patients. Since a transient phase may precede the full-blown clinical manifestation of AD, we examined the role of OCT also in sensing RNFL thickness changes in MCI. We found that the OCT is a well-suited paraclinical methodology to assess RNFL thickness in both AD and MCI disorders.

Despite many advances in the understanding of its pathophysiology, AD is still difficult to diagnose, chiefly because: a) diagnosis is mainly based on psychometric assessments by a multidisciplinary team (neurologists, psychiatrists, and psychologist), b) because physical and neurological examinations are not specific for this disorder, and c) because, brain physiological senescence processes may mask the subtle pathological neurodegenerative processes leading to AD. Because of inter- and intra-individual variability, neurophysiological and neuroimaging tests have not yet been considered as criteria on which to base a diagnosis. In fact, neurophysiological as well as other paraclinical tests are recommended only on suspicion of secondary causes of dementia as part of the differential diagnosis of AD. A confirmation of the AD diagnosis is possible only with *post mortem* histopathology. Nonetheless, the last few decades have seen the use of structural and functional techniques as potential biomarkers that might also identify factors that may predispose individuals to AD.

The OCT technique for measurement of the peripapillary RNFL, the macular thickness and volume, has been used as useful tool for the detection of significant retinal changes in patients and these measures roughly correlate with the severity of the disease.

Results from our meta-analysis based on 11 studies, suggested that AD patients are likely to have a reduced RNFL thickness as assessed by OCT. This reduction in RNFL thickness, as observed in most studies on AD patients, was significantly greater than that observed in the age-matched controls and thus cannot be exclusively ascribed to aging (see a synopsis of published OCT studies in AD in Table 1). Further meta-analysis showed a uniform significant decrease in RNFL thickness in each of the four retinal quadrants, suggesting that whatever factors cause the degenerative process in AD progression, affect the entire retinal layer.

Although little is known about the neurobiological basis of the physiological and structural changes that occur in the brain of AD patients, some histological and morpho-functional studies point to the concept that the same neurodegenerative processes, which affect the brain, may also affect the nerve fiber layer of the retina, as an integrated part of the nervous system.

In animal models, expressing mutant forms of amyloid precursors, and histological studies on post mortem human AD eyes, researchers have observed various retinal pathological changes, such as depletion of axons in the optic nerves and extensive retinal ganglion cell loss [20,21,50,51]. These changes were accompanied by accumulation within the retina and its microvasculature of toxic aminoacids classically described in the degenerative processes of AD, such as fibrillar tau and Aβ aggregates and specific signs of neuroinflammation [52–57].

In investigations using the optic nerve analyser, a higher proportion of AD patients than age-matched healthy subjects showed signs of optic neuropathy and this manifested as optic disc atrophy, pathologic optic disc cupping, and thinning of the neuroretinal rim and of the RNFL [58,59].

In agreement with post mortem studies, OCT data studies indicate a significant decline in peripapillary RNFL and changes in macular thickness and volume that is progressive from MCI to AD eyes. Parisi and colleagues first used OCT to study a group of AD patients and compared them with a group of age-matched controls. In AD patients, OCT results showed a reduced thickness of the retina NFL overall and in each quadrant examined they found involvement of the neuroretinal tissue in AD [31,32]. The mean RNFL thickness was confirmed to be reduced in AD patients by several independent groups [33–36]. Most studies observed a significant reduction of RNFL thickness in all quadrants [31,32,36,44], but predominantly in the superior [34,35,37,43,60] and inferior quadrants [34,37]. The retinal thinning in AD patients was confirmed despite the different commercially available OCT devices used [36,49].

OCT studies in AD patients showed RNFL thickness reduction that was directly proportional to abnormalities in pattern electroretinogram, reflecting neuronal degeneration in the retinal ganglion cell layer [31,32], not related to changes in cortical visual evoked responses, and hence probably not a consequence of retrograde degeneration [60]. Both positive [60,61] and absent [33,35,37,43,62] correlation was observed between the reduced total macular volume in patients with AD and the severity of the disease as assessed by the mini mental state examination questionnaire (MMSE) [60,61]. Unfortunately, it was not possible to make a subgroup analysis for this due to lack of power and high variability of the data.

OCT is be a useful tool for evaluating the progression of neurodegenerative processes that lead to AD. Based on 3 studies, our present meta-analysis showed a significant RNFL reduction in MCI patients as well compared with age-matched healthy controls. Ancillary quadrant meta-analysis in MCI revealed a significant reduction in RNFL thickness for each quadrant, especially in the inferior and nasal, with the exception of the superior, where the reduction only approached the significance level (p = 0.06). This lack of significance can be ascribed to the lack of statistical power due to small amount of patients enrolled. Further studies in a large cohort of patients are needed in order to clarify the exact degree of each quadrant involvement in MCI. Few studies compared MCI versus AD patients and healthy aged people. Kesler et al. [37] observed that the mean RNFL was significantly thinner in both AD and MCI patients groups compared to controls, and that the MCI group fell in between the other two groups. This difference was particularly prominent in the inferior quadrant, whereas AD patients had significantly thinner retinal NFL values also in the superior quadrant [37].

Finally, present meta-analysis cannot overcome certain limitations of the literature. First, the total number of patients is smaller than we would expect to generalize our results, although our cohort was sufficient to disclose strong statistical significance. Second, the variable assortment of OCT instruments (we counted 7 different apparatus) used across studies prevented us doing examination of whether differences in RNFL thickness were attributable to different types of OCT. The latter is of particular relevance for the reproducibility of OCT results in low compliance patients, considering, among all, the intrinsic between apparatus variability in centring procedure around the optic disc, eye tracking system, and in length of examination time.

## Conclusions

Overall, the results of this meta-analysis showed that RNFL thickness decreased in all quadrants in AD patients. These findings strongly suggest that degeneration of retinal ganglion cells should be added to the constellation of neuropathologic changes found in patients with AD on the one hand, and that RNFL thickness can be used to distinguish AD patients from normal ageing, on the other. Moreover, the meta-analysis data also revealed that OCT can be useful to detect early RNFL abnormalities in MCI patients. Whether the subgroup of MCI patients with thinner RNFL have a higher annual incidence of conversion to AD [63] remains to be determined in an appropriately designed study. This is of particular interest in view of using OCT as a paraclinical test with prognostic value.

Future research in this subject area will surely provide a better assessment of the specificity of these OCT findings in AD, and their potential correlation with disease severity. We will see RNFL thickness measurements in patients with other dementias as an aid to diagnosis and we will see whether the RNFL can be used as a surrogate for magnetic resonance imaging (MRI) measurements of the brain.

## Supporting Information

S1 Fig PRISMA flow diagram of the search and study selection process. (TIFF)
