# Supplementary figures and images for: Optical Coherence Tomography in Alzheimer’s Disease: A Meta-Analysis
Source: PLoS One. 2015 Aug 7;10(8):e0134750. doi: 10.1371/journal.pone.0134750 (PMC4529274; doi:10.1371/journal.pone.0134750)

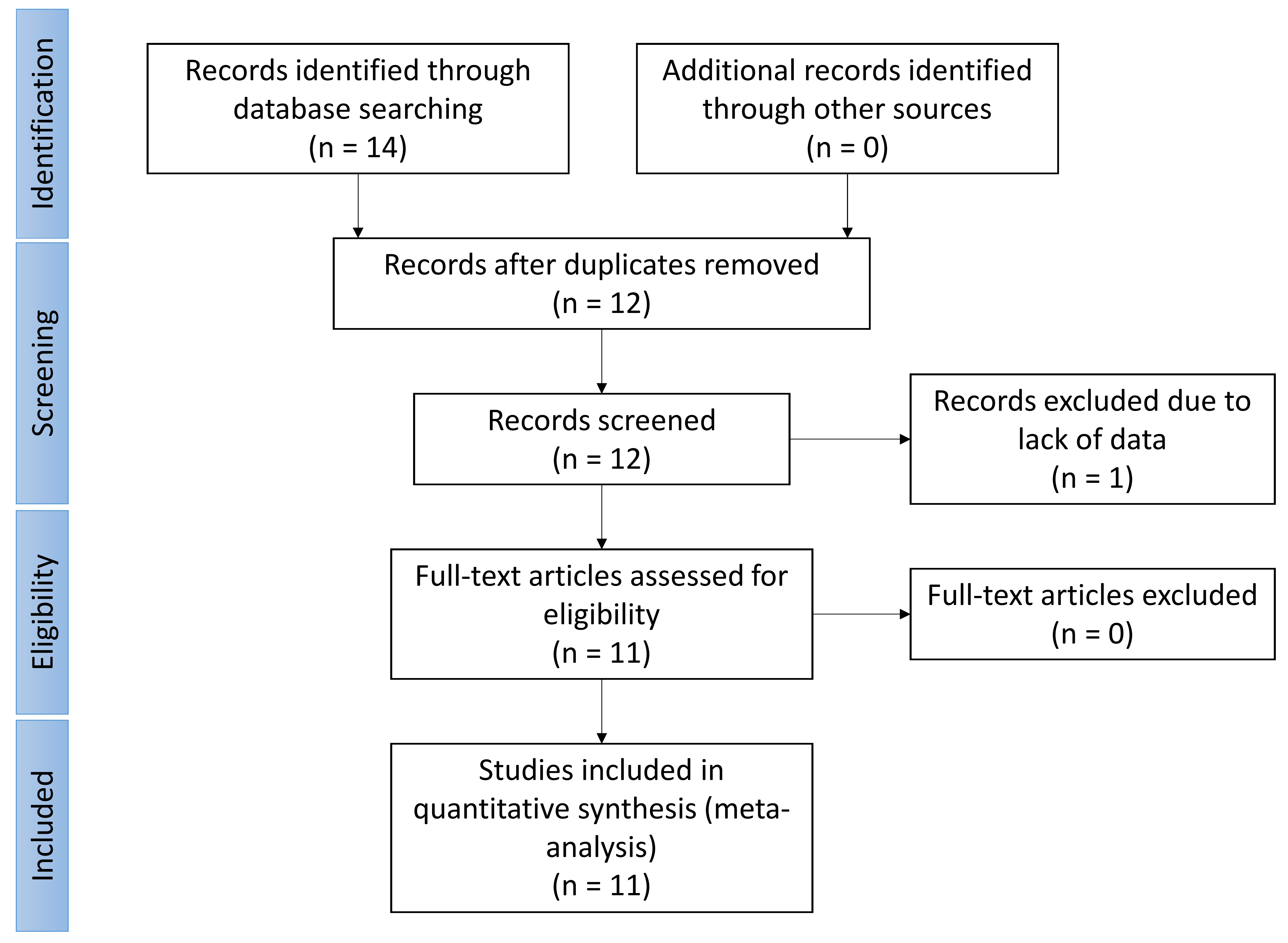

Supplement: S1 Fig — (TIFF) [file pone.0134750.s001.tiff]
